# Endovascular therapy for iatrogenic occlusion of the popliteal artery after complex total knee arthroplasty: A case report

**DOI:** 10.1016/j.jvscit.2024.101695

**Published:** 2024-11-26

**Authors:** Imam T.P. Ritonga, Giuseppe Asciutto, Kevin Mani, Yousef Shehada, Marco Virgilio Usai

**Affiliations:** a Department of Vascular Surgery, St Franziskus Hospital, Münster, Germany; b Section of Vascular Surgery, Department of Surgical Sciences, Uppsala University, Uppsala, Sweden

**Keywords:** ART, Covered stent, Endovascular therapy, Iatrogenic arterial injury, Occlusion, TKA

## Abstract

Iatrogenic arterial injury is an infrequent but limb-threatening complication of total knee arthroplasty (TKA). Open surgical reconstruction may not always be feasible or optimal, particularly in patients who have recently just undergone complex TKA procedures. In this report, we describe the treatment of a patient who developed popliteal artery occlusion following a complex TKA procedure performed the previous day. Although endovascular advancement allows comprehensive endovascular treatment of acute limb ischemia, their application in the context of iatrogenic arterial injury after TKA is limited, with only a few cases documented. This case is notable due to the presence of both penetrating trauma to the popliteal artery and subsequent thrombosis of the artery. These complications were successfully managed entirely through endovascular therapy, employing AngioJet rheolytic thrombolysis and the implantation of a covered stent. This case adds to the growing body of evidence supporting the use of endovascular techniques for managing this rare and complex complication of TKA.

Total knee arthroplasty (TKA) is among the most frequently performed surgical procedures globally. In the United States alone, 480,958 TKA procedures were conducted in 2019, with projections indicating this number will rise to 1.2 million by the year 2040.^1^ In Germany, the annual number of TKA procedures reaches approximately 193,000.^2^ Like other surgical interventions, TKA is not devoid of risks and complications, including arterial injury. Iatrogenic arterial injury (IAI) primarily results from penetrating trauma, with a smaller proportion due to blunt trauma during the procedure, leading to bleeding, hematoma, occlusion, and ischemia.^3^ The incidence of IAI involving the popliteal artery is relatively low, ranging from 0.017% to 0.51%. However, it can result in ischemic nerve injury and irreversible ischemia, with amputation rates between 5.9% and 42%, and mortality rates as high as 7%.^4^^,^^5^ Therefore, prompt intervention is essential to address IAI in the popliteal artery and mitigate these severe consequences. Open surgical repair (OSR) has been traditionally seen as the treatment of first choice.^3^^,^^5^ However, OSR may not always be feasible, especially in patients who have just undergone complex TKA procedures. In such cases, an alternative or complementary endovascular approach can be of value.

## Case description

**Fig 1:**
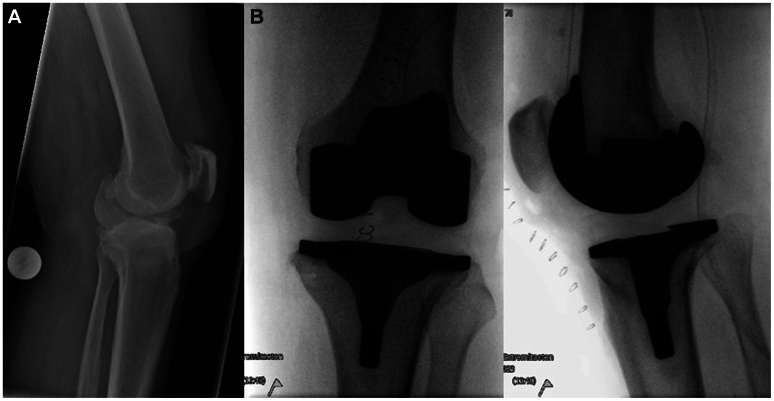
Preoperative **(A)** and intraoperative **(B)** imaging of the knee during the total knee arthroplasty (TKA).

**Fig 2:**
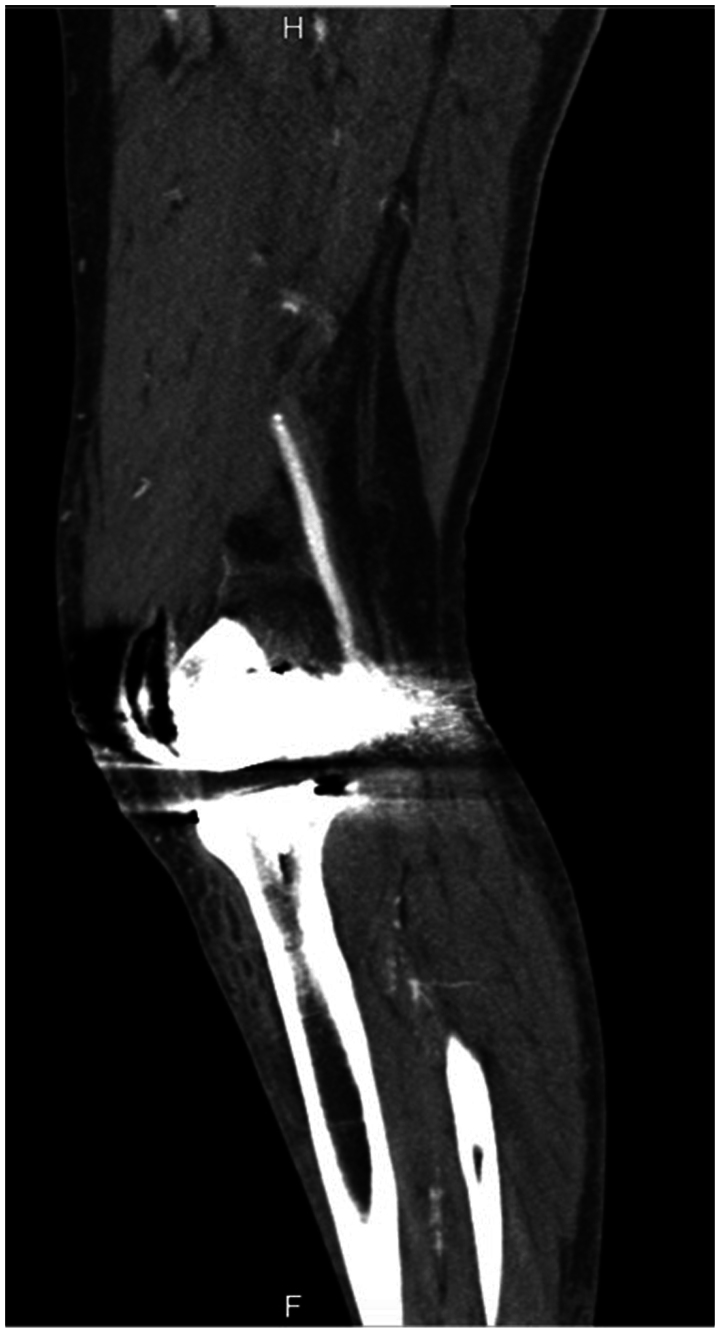
Computed tomography image following the total knee arthroplasty (TKA) showing the occlusion of popliteal artery (PA).

A 73-year-old male patient was initially referred to our department from the orthopedics unit after undergoing a TKA on the left side the previous day. The procedure was highly challenging due to the fixed varus contracture and complete imbalance of the capsular ligament apparatus, as well as severe synovitis (Fig 1). On the following day, the patient developed significant swelling of the knee joint and progressive weakness of dorsiflexion of the foot, clinically raising suspicion of compartment syndrome. Blood tests also showed increased creatine kinase levels of 10,616 U/L. Emergency computed tomographic angiography revealed a soft tissue hematoma in the left popliteal fossa, without clear evidence of active bleeding. Furthermore, the popliteal artery (PA) was occluded at the height of the knee joint. The fibular artery (FA) and the posterior tibial artery (PTA) refilled through collaterals, whereas the anterior tibial artery (ATA) was occluded (Fig 2). The patient has no known history of vascular disease and did not take any antiplatelet or anticoagulant until the time of the operation. After explaining the computed tomography results and obtaining consent from the patient, the operation was immediately performed.

**Fig 3:**
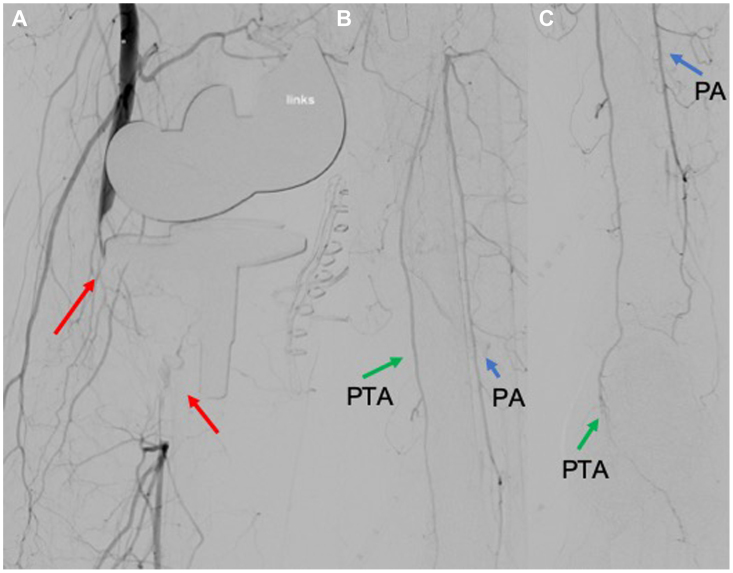
Intraoperative angiography. **A,** Complete thrombotic occlusion of the popliteal artery (*PA*) and anterior tibial artery (ATA) (*red arrow*), **B** and **C,** Refilling of the fibular artery (FA) (*blue arrow*) and the posterior tibial artery (*PTA*) (*green arrow*), whereas the ATA was occluded.

The patient underwent an immediate fasciotomy through the orthopedic surgeons, followed by the initiation of a negative pressure wound therapy dressing (3M V.A.C. Therapy). Thereafter, an endovascular approach was performed under local anesthesia. A crossover 8 Fr Destination sheath (Terumo) was advanced from the right common femoral artery to the left superficial femoral artery. Intraoperatively, 5000 IU heparin was given. The intraoperative angiography showed an occlusion of the PA at the height of the knee joint. The ATA was not visualized, whereas the PTA and the FA showed a significant delayed contrast filling peripherally. The foot was perfused mainly through the PTA (Fig 3).

Recanalization was achieved using a 0.035-inch Glidewire Advantage guidewire (Terumo) and a 135-cm quick-cross support catheter (Philips-Spectranetics). A 6 Fr AngioJet rheolytic thrombectomy system (Boston Scientific) was advanced through the occluded PA. Local thrombolysis with 10 mg alteplase diluted in 500 mL saline infusion was administered through the AngioJet catheter. Control angiography showed restored flow to the PTA and the FA. The PA was open with residual marginal thrombus.

**Fig 4:**
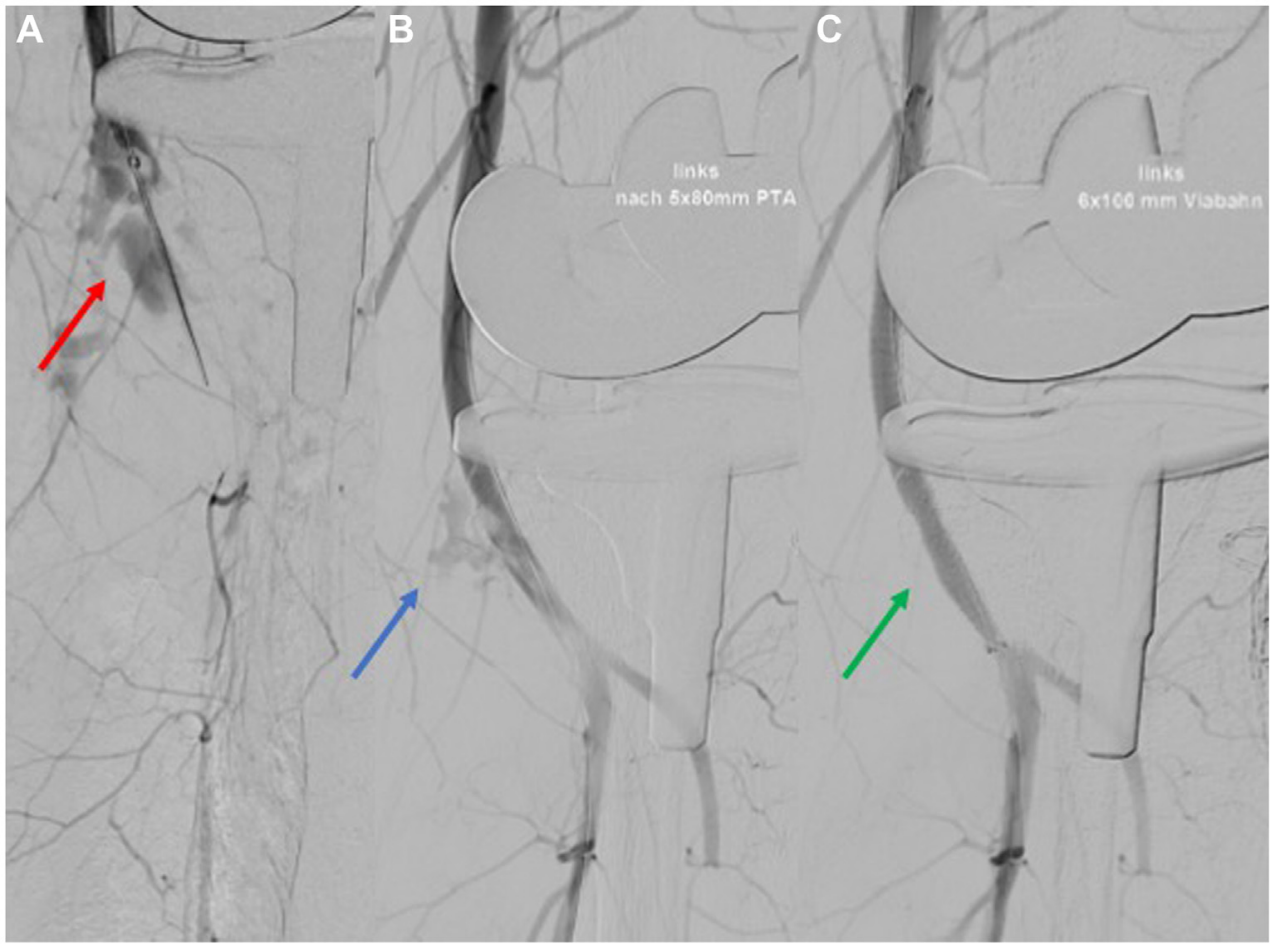
Intraoperative angiography. **A,** Active bleeding from the popliteal artery (PA) (*red arrow*). **B,** Persistent bleeding after prolonged inflation of an angioplasty balloon (*blue arrow*). **C,** Control angiography after the implantation of the Viabahn (*green arrow*).

**Fig 5:**
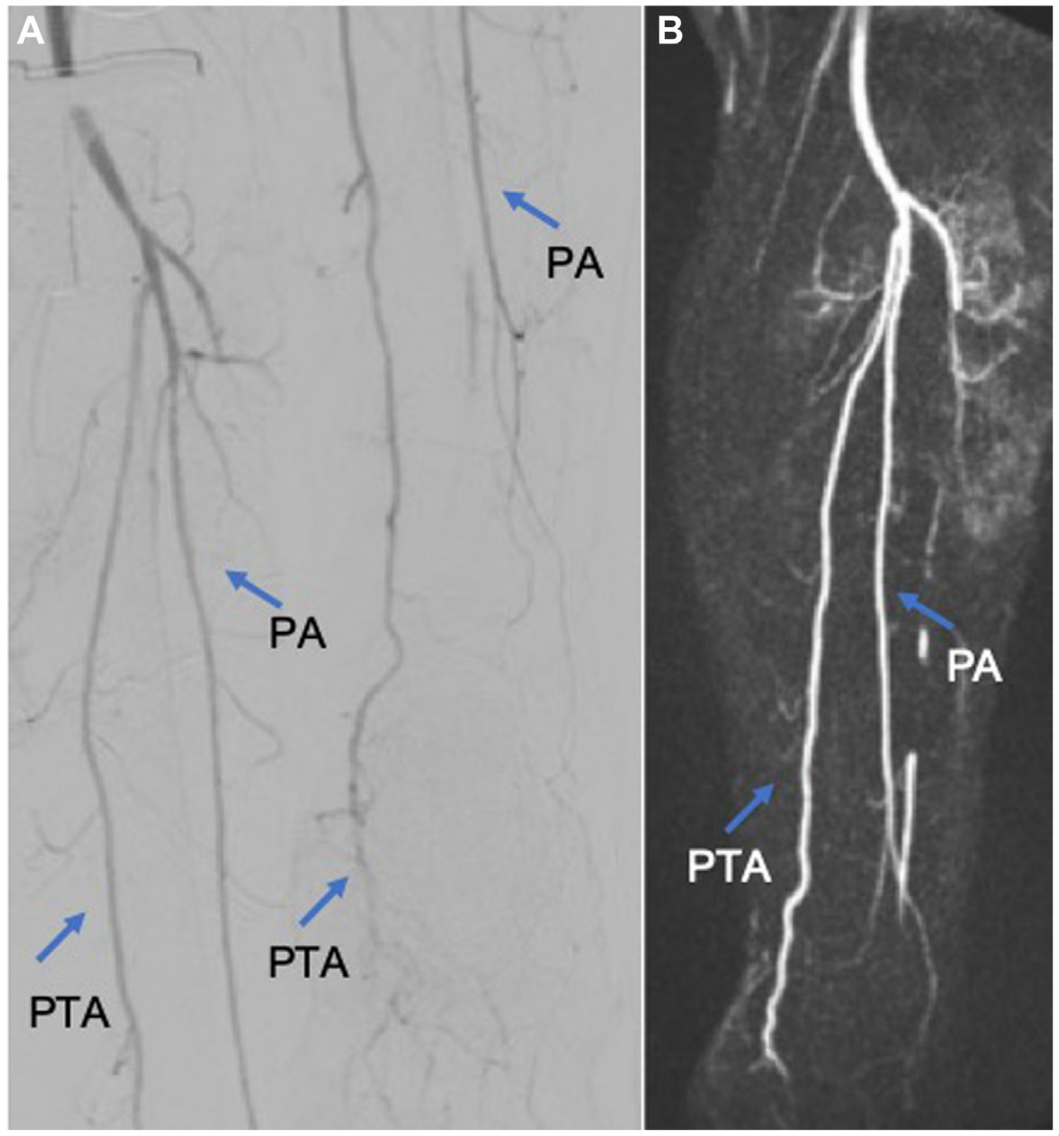
The completion angiography **(A)** and postoperative magnetic resonance angiography **(B)** both demonstrated patency of the popliteal artery (*PA*), fibular artery (FA), and posterior tibial artery (*PTA*) (*blue arrow*).

However, during this process, increased blood accumulation was observed in the previously applied vacuum sponge. The follow-up angiography showed an active bleeding from the PA (Fig 4). To address this, we did a 5-minute prolonged inflation using a non-compliant 5-mm angioplasty balloon. A persisting bleeding at the control angiography resulted in the deployment of a 5 × 100 mm Viabahn stent graft (W. L. Gore & Associates). Subsequent angiography confirmed a patent PA with no signs of bleeding. The FA and PTA, as well as the plantar arch, were patent, whereas the ATA was still occluded at the origin (Fig 5, *A*). Clinically, the foot looked well-perfused with a fast capillary refilling. The procedure was concluded by closing the puncture site with an 8 F Angio-Seal (Abbott Vascular). Heparin was administered postoperatively at 400 IU/hour for 24 hours using a perfusor pump.

Postoperatively, the distal PTA was palpable, and the sensory and motor functions of the left leg were regained. Creatine kinase value peaked 1 day after the procedure (11,532 U/L). On the second day, it began to show a decreasing graph (9719 U/L) until it reached normal at the time of discharge (286 U/L). Postoperative magnetic resonance angiography (Fig 5, *B*) confirmed satisfactory results with patency of the PA and the PTA. In the further course, the wounds were regularly debrided surgically, treated with temporary negative pressure wound therapy dressing, and were later closed successively. However, during the inpatient stay, the patient had COVID-19 infection and then also an ischemic stroke, which occurred approximately 1 month after thrombolysis intervention. These two factors caused the length of his hospital stay to be extended. The patient was transferred on the 36th day after the initial vascular intervention to the neurological department at another hospital for further observation following a stroke. By the time of transfer, the patient showed no further complications on his leg.

The patient provided written consent for the publication of this case report.

## Discussion

The incidence of IAI involving the PA is actually relatively low, but it can result in ischemic nerve injury and irreversible ischemia, with amputation rates ranging from 5.9% to 42%. OSR is traditionally considered the treatment of first choice for popliteal IAI after TKA. However, it may not always be feasible or optimal, especially in patients who have just undergone complex TKA procedures. Notably, redo-TKA procedures have been associated with double the rate of arterial injuries.^4^

We present a case of an iatrogenic lesion of the PA after a complex TKA procedure treated endovascularly. The TKA was particularly challenging due to a fixed varus contracture, complete imbalance of the capsular ligament apparatus, severe synovitis, and a high tendency to bleeding. Once the diagnosis of PA occlusion was confirmed, this information was crucial in determining the procedural approach. The actual case was particularly challenging due to the presence of a primary iatrogenic lesion causing a bleeding and, consequently, a thromboembolic occlusion.

Addressing these two problems, the bleeding and the occlusion, could be difficult with open approaches. The usually limited anatomical vessel exposure in the popliteal fossa, as well as the presence of a hematoma, forced us to think of an alternative solution.

In this case, the use of the AngioJet rheolytic thrombectomy system also showed results that were actually satisfactory because it could initially reopen blood flow in the PA, FA, and PTA. The subsequent angiography evidence of bleeding from the PA most likely indicates the actual primary problem that occurred, namely penetrating trauma to the PA that may have occurred since the TKA operation, which then resulted in occlusion due to the consequences of primary fibrinolytic hyperactivity and hypercoagulability, as well as pressure due to hematoma accumulation.

Despite this sudden finding, immediate bleeding control was achieved without resorting to open surgery, which would have been particularly challenging in a difficult post-TKA scenario. Bleeding was initially controlled using plain old balloon angioplasty, but a covered stent was eventually deployed after persistent bleeding was observed.

Although the intraoperative results are promising, the issue of the patency of a stent graft placed in the PA at the height of the knee joint must be addressed. The Viabahn stent graft is placed in this region mostly in case of popliteal aneurysm. In this group of patients, the reported patency of 100% at 1 year and 90% at 2 years is surely promising.^6^ In the present case, the patient has been under strict ultrasound surveillance, with the last control done 2 months postoperatively showing a patent stent graft. Further controls twice a year are planned. Another important aspect is that of the anticoagulation/antiplatelet regime to use in cases similar to the one we present. We opted in the present case for dual antiplatelet therapy using aspirin (100 mg/day) and clopidogrel (75 mg/day) for at least 3 months. Subsequently, single antiplatelet therapy with aspirin (100 mg/day) is planned to be prescribed for the patient’s lifetime.

## Conclusion

IAI affecting the popliteal artery following TKA is an uncommon but potentially serious complication that can threaten limb viability. In cases where open surgery is challenging, endovascular treatment offers an effective therapeutic option with satisfactory outcomes. A strict follow-up is paramount and can be easily performed with ultrasound.

## Funding

None.

## Disclosures

None.
